# Biomarkers in Rheumatoid Arthritis, what is new?

**Published:** 2016

**Authors:** BI Gavrilă, C Ciofu, V Stoica

**Affiliations:** * Department of Internal Medicine and Rheumatology, “Dr. I. Cantacuzino” Clinical Hospital, Bucharest, Romania; “Carol Davila” University of Medicine and Pharmacy, Bucharest, Romania

**Keywords:** rheumatoid arthritis, biomarkers, disease activity, biologic therapy

## Abstract

Rheumatoid arthritis (RA) is a chronic inflammatory disease with autoimmune pathogenesis. It affects mainly small joints (of the hands and feet) and has many systemic manifestations.

Studying biomarkers in rheumatology intensely appeared from the need to understand the mechanisms underlying some rheumatic diseases. Discovering new biomarkers with key roles in various stages of evolution, remains a subject of interest for RA.

Currently, according to the EULAR 2010 criteria, the rheumatoid factor (RF) and the anti-cyclic citrullinated peptide (anti-CCP) are used for RA diagnosis. Since 2010, new biomarkers were discovered and proved useful in identifying RA in early stages.

For a more rigorous management of these cases, one of the key steps in the evolution of patients with RA is to recognize and distinguish the more aggressive forms of the disease through prognostic biomarkers.

“Treat to target” recommends the use of 3 composite scores to monitor the evolution of the disease: disease activity score (DAS 28), simple disease activity index (SDAI) and clinical disease activity index (CDAI), but, a new test was developed which better monitors the disease activity.

The introduction of biological therapies has revolutionized the treatment of RA. Despite these advances, 20-40% of the patients are declared nonresponders to at least one of the therapies. The patient exposure to the potential side effects and high costs requires the discovery of a biomarker that could identify those who can benefit from the pretreatment of a certain therapy.

**Abbreviations:** RA = rheumatoid arthritis, RF = rheumatoid factor, DAS 28 = disease activity score, SDAI = simple disease activity index, CDAI = clinical disease activity index, ACR = American College of Rheumatology, EULAR = European League against Rheumatism, anti-CCP = antibodies against cyclic citrullinated proteins, anti-MCV = mutated citrullinated vimentin antibodies, anti-CarP = antibodies against carbamylated proteins, MBDA = multi biomarker disease activity test, COMP = cartilage oligomeric matrix protein, ADAs = antidrug antibodies, CDA = clinical disease activity index, SDAI = simplified disease activity index, ESR = erythrocyte sedimentation rate, CRP = C reactive protein, SAA = serum amyloid A, VCAM-1 = vascular cell adhesion molecule-1, IL-6 = interleukin-6, TNF-R1 = tumor necrosis factor receptor 1, EGF = epidermal growth factor, VEGF-A = vascular endothelial growth factor A

## Introduction

Rheumatoid arthritis (RA) is a chronic inflammatory disease with autoimmune pathogenesis, characterized by joint involvement (that leads to deforming and destructive arthritis), and multiple systemic manifestations.

The etiology of RA remains unknown, multiple mechanisms being involved in the physiopathogenic chain. The heterogeneity of clinical manifestations and variability of therapeutic response demonstrates the complexity of this disease [**1**].

The progress in understanding the pathogenesis of RA processes increased the interest in studying the biomarkers involved in different stages of the disease, new biomarkers being identified.

**Fig. 1:**
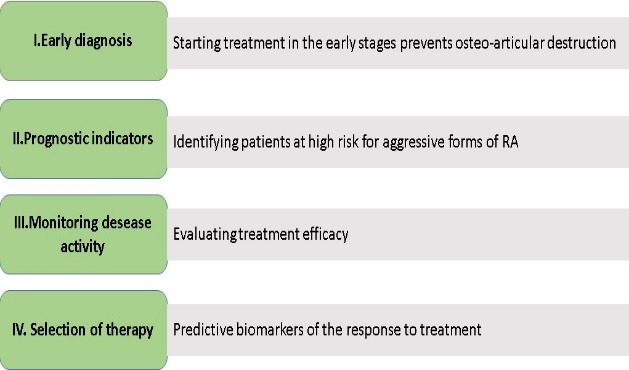
Need for Biomarkers

In its evolution, there are several key stages and their proper management may influence the further progression (**Fig. 1**).

**Fig. 2:**
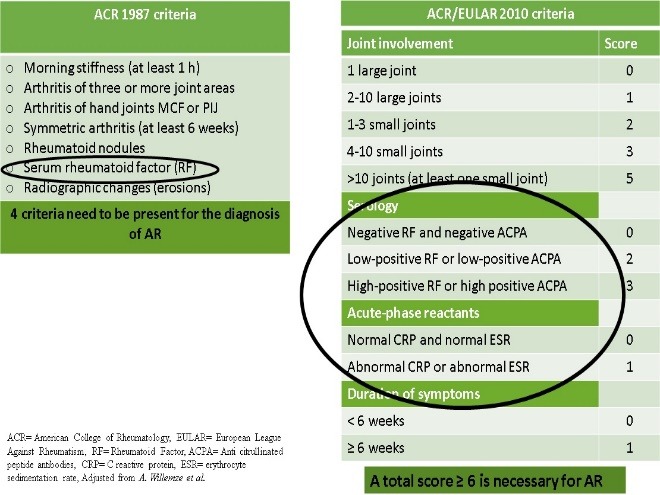
ACR 1987 vs. ACR/ EULAR 2010 criteria for the diagnosis of RA

The major role of biomarkers can be objectified by comparing the diagnostic criteria. The only ACR 1987 criteria biomarker is the rheumatoid factor (RF). The new ACR/ EULAR 2010 criteria for the early diagnosis of RA use four serological tests (**Fig. 2**).

**I. Diagnostic biomarkers**

The new concept “window of opportunity” shows that the RA identification in the early stages is essential to prevent erosion and to stop the progression of radiologic changes. In this context, the attention paid to the identification of biomarkers with a diagnostic role in the early stages of the disease is still a subject of great interest [**2**,**3**].

**Fig. 3:**
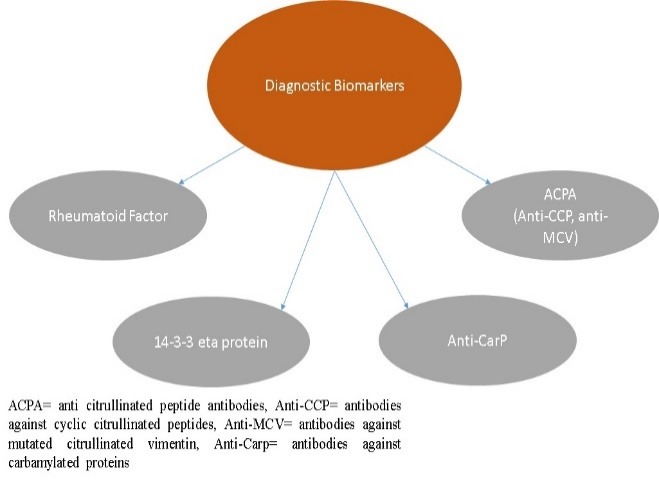
Diagnostic biomarkers for RA

Currently, the ACR/ EULAR 2010 criteria for the RA diagnosis use the rheumatoid factor (RF) and antibodies against cyclic citrullinated proteins (anti-CCP). Besides them, other diagnostic biomarkers that can help the early diagnosis of RA were identified (**Fig. 3**).

Vimentin is a protein that can be citrullinated, a reaction mediated by peptidyl arginine deiminase with the formation of anti-vimentin antibodies. To improve the quality of the test and starting from the hypothesis that additional changes may influence vimentin antigenicity, a mutation was performed in which arginine residues are replaced with glycine causing the formation of mutated citrullinated vimentin antibodies (anti-MCV).

A meta-analysis from 2010 that included 14 studies, in which the anti-MCV and anti-CCP for the RA diagnosis were tested, concluded that there is no difference between the two tests. Thus, the anti-MCV may be a test of line 2, used in patients suspected of RA, but with anti-CCP and RF negative [**4**].

14-3-3 eta protein belongs to the family of 14-3-3 proteins that consists of 7 isoforms, it is localized intracellularly, being externalized in the inflammatory process where it can be citrullinated. The 14-3-3 proteins act like chaperonins [**5**].

In a study conducted on 619 subjects, 14-3-3 eta protein sensitivity and specificity for RA was 77% and 93% respectively. In the early stages of the disease, the determination of protein 14-3-3 eta along with RF and anti-CCP increases the diagnostic rate from 72% (RF + anti-CCP) to 78% (RF + anti-CCP + 14-3-3eta ) [**6**].

In 2011, antibodies against carbamylated proteins (anti-CarP) were found in the serum of patients with RA. In a study performed on 2086 patients with early RA, anti-CarP, anti-CCP (generation II) and RF (Ig M) were tested. Results showed a sensitivity of 44% and a specificity of 89% for the anti-CarP, compared with the anti-CCP sensitivity and specificity of 54% and 96% or RF who had 59%, respectively 91% [**7**].

**II. Prognostic biomarkers**

RF presence and high titers were correlated with an increased risk of developing RA. The risk may increase even 26 times if the initial titers of RF are > 100 IU/ ml [**8**]. The presence of the IgA isotype is associated with extra-articular manifestations [**9**].

Patients with RF present, usually develop more aggressive forms of the disease, with a more severe functional impairment. Rheumatoid nodules are a frequent manifestation for the presence of RF in the serum of patients [**10**].

In a study that included 279 patients with early forms of RA, who were followed for five years by clinical examination, serological and imaging tests, the presence of anti-CCP at diagnosis was associated with a more important radiological progression and severe forms of disease [**11**].

The presence of other biomarkers was associated with more severe forms of RA, such as anti-MCV, 14-3-3 eta protein, but these tests are not commonly used and need to be implemented in guidelines [**12**,**13**].

**III. Biomarkers for the monitorization of the disease activity**

In the “treat to target” recommendations, 3 composite scores for the monitorization of the disease evolution are used: disease activity score (DAS 28), simple disease activity index (SDAI) and clinical disease activity index (CDAI). The disadvantage of these scores is the degree of subjectivity of some of the criteria. Moreover, a significant proportion of the patients with negative inflammatory tests, still have active disease [**14**-**16**].

For a better monitorization of the disease activity, a test that includes several biomarkers under the name “multi-biomarkers disease activity test (MBDA)” has been developed” (**Table 1**) [**17**].

Depending on the values of this score, the disease activity can be classified into mild (1-28), moderate (29-43) or severe (>44). The usefulness of this score determined the commercialization of Vectra DA test, which is now available for clinicians.

**Table 1:** Parameters for the monitorization of the disease activity

PARAMETERS	DAS28	CDAI	SDAI	MBDA
TENDER JOINTS	√	√	√	Acute phase reactants: **CRP, SAA**
SWOLLEN JOINTS	√	√	√	Adhesion molecules: **VCAM-1 **
PATIENT GLOBAL ASSESSMENT OF DISEASE ACTIVITY	√	√	√	Cytokines and related proteins: **IL-6 and TNF-RI**
CLINICIAN GLOBAL ASSESSMENT OF DISEASE ACTIVITY		√	√	Matrix metalloproteinases (MMP): **MMP-1 and MMP-2 **
ESR or CRP	√		√	Human cartilage glycoprotein 39
				Growth factors: **EGF and VEGF-A**
				Hormones: **leptin and resistin**
DAS 28 = disease activity score, CDA = clinical disease activity index, SDAI = simplified disease activity index, ESR = erythrocyte sedimentation rate, CRP = C reactive protein, SAA = serum amyloid A, VCAM-1 = vascular cell adhesion molecule-1, IL-6 = interleukin-6, TNF-R1 = tumor necrosis factor receptor 1, EGF = epidermal growth factor, VEGF-A = vascular endothelial growth factor A				

**IV. Predictive biomarkers of the response to biologic therapy**

The introduction of biological therapies in the management of RA represented a major step forward for a better control of the disease. A better understanding of the pathophysiological mechanism underlying AR allowed the development of biological therapies that act at different levels of pathogenic chain.

**Fig. 4:**
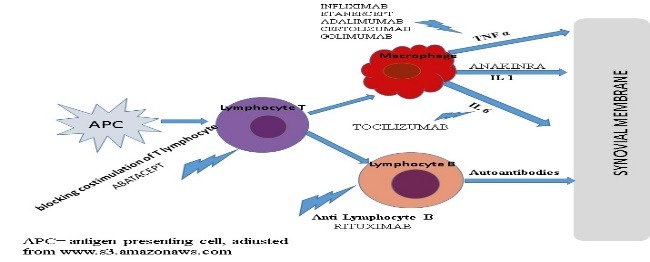
Site of action of biological agents

Therapies currently available are anti TNF α: Infliximab (IFX), Adalimumab (ADA), Etanercept (ETA) Certolizumab (CER), Golimumab pegol (GOL), anti-lymphocyte B - Rituximab (RTX), interleukin 1 antagonists receptor Anakinra (ANA), interleukin 6 receptor antagonists - Tocilizumab (TCZ), blocking costimulation of T lymphocyte - Abatacept (ABA) (**Fig. 4**).

Despite this progress, studies reported that 20-40% of the patients were declared nonresponders after starting a biologic therapy [**18**].

High costs and patient exposure to severe adverse reactions (ex. infections) determined the need to identify biomarkers that can distinguish pretreatment responders vs. nonresponder patients. Over time, several potential biomarkers were tested and identified with this role, but none was adopted in practice. 

Regarding this RF, anti-CCP [**19**], anti-MCV [**20**,**21**], 14-3-3 eta protein [**22**,**23**], cartilage oligomeric matrix protein (COMP) [**24**] and serum calprotectin were tested [**25**] (**Table 2**). Immunogenicity was also analyzed.

Cartilage oligomeric matrix protein (COMP) is a specific serological marker, which evaluates the articular cartilage degradation and its turnover. It is detectable in the blood and synovial fluid [**24**].

Serum calprotectin is a protein that has the ability to bind calcium, belonging especially to leukocytes, but can also be found in monocytes or macrophages. High levels of calprotectin can appear in various inflammatory conditions [**25**].

Survivin, a tumoral biomarker, which belongs to the family of inhibitors of apoptosis, has been reported in patients with RA [**26**].

**Table 2:** Biomarkers for the prediction of the response to biologic therapy

Biomarker	Presence(P)/Absence(A)	Predictive role	Therapy
FR,Anti-CCP	Neither P or A	-	Anti-TNF
FR,Anti-CCP	P	++	RTX
Anti-MCV	Neither P or A	-	Anti-TNF
Anti-MCV	P	+	RTX
14-3-3eta	A or low levels	+	TCZ, anti-TNF
COMP	A or low levels	+	ADA
Calprotectin	P	+	ADA,IFX,RTX
Survivin	A or low levels	+	IFX
Rheumatoid factor (FR), Antibodies directed to cyclic citrullinated peptides (Anti-CCP), Antibodies against mutated citrullinated vimentin (Anti-MCV), Cartilage oligomeric matrix protein (COMP) failed to predict (-),Confirmed by small studies, require testing on larger groups (+) confirmed by several studies (++) Anti tumor necrosis factor (Anti-TNF),Adalimumab (ADA), Infliximab (IFX) Rituximab (RTX), Tocilizumab (TCZ)			

The immunogenicity is one possible cause of non-responsiveness to a biological treatment process, which consists in the appearance of anti-drug antibodies (ADAs).

One of the strategies proposed to decrease the immunogenicity of biological agents is their association with Methotrexate. It is not well understood how it reduces the production of ADAs, perhaps by inhibiting lymphocytes [**27**].

Due to the unique structure between the anti-TNF agents, ETA is less immunogenic. There are rare cases for the appearances of ADAs against it [**28**].

## Conclusions

The identification of new biomarkers with a real clinical utility remains a major topic of interest in RA.

Along with the RF and anti-CCP, the determination of anti-MCV proved to be a useful tool in the diagnosis of RA in early stages.

A new biomarker joined them, the 14-3-3 eta protein, which has been incorporated into the IdentRA test, together with the anti-CCP and RF for the diagnosis of RA. It can also be used individually, under the name JOINTstat. These two tests are now available in the US and Canada.

For a more effective monitoring of the disease, MBDA score has been developed. It uses the determination of some biomarkers involved in the pathogenesis of the RA chain, and it is available for clinicians under the name VectraDA.

The identification of a biomarker that could detect the pretreatment for patient responders vs. nonresponders remains an unsolved issue. A major candidate could be the 14-3-3 eta protein, but studies on larger groups of patients and for longer periods need to confirm this value.
